# Discrimination of the Activity of Low-Affinity Wild-Type and High-Affinity Mutant Recombinant BoNT/B by a SIMA Cell-Based Reporter Release Assay

**DOI:** 10.3390/toxins14010065

**Published:** 2022-01-17

**Authors:** Frank Neuschäfer-Rube, Andrea Pathe-Neuschäfer-Rube, Gerhard P. Püschel

**Affiliations:** Department of Nutritional Biochemistry, Institute of Nutritional Science, University of Potsdam, 14469 Potsdam, Germany; dr.apnr@gmail.com (A.P.-N.-R.); gpuesche@uni-potsdam.de (G.P.P.)

**Keywords:** cell-based assay, genetically modified BoNT, BoNT/B uptake

## Abstract

Botulinum neurotoxin (BoNT) is used for the treatment of a number of ailments. The activity of the toxin that is isolated from bacterial cultures is frequently tested in the mouse lethality assay. Apart from the ethical concerns inherent to this assay, species-specific differences in the affinity for different BoNT serotypes give rise to activity results that differ from the activity in humans. Thus, BoNT/B is more active in mice than in humans. The current study shows that the stimulus-dependent release of a luciferase from a differentiated human neuroblastoma–based reporter cell line (SIMA-hPOMC1-26-Gluc) was inhibited by clostridial and recombinant BoNT/A to the same extent, whereas both clostridial and recombinant BoNT/B inhibited the release to a lesser extent and only at much higher concentrations, reflecting the low activity of BoNT/B in humans. By contrast, the genetically modified BoNT/B-MY, which has increased affinity for human synaptotagmin, and the BoNT/B protein receptor inhibited luciferase release effectively and with an EC50 comparable to recombinant BoNT/A. This was due to an enhanced uptake into the reporter cells of BoNT/B-MY in comparison to the recombinant wild-type toxin. Thus, the SIMA-hPOMC1-26-Gluc cell assay is a versatile tool to determine the activity of different BoNT serotypes providing human-relevant dose-response data.

## 1. Introduction

Botulinum toxins (BoNTs) are highly potent neurotoxins that act by inhibiting the fusion of synaptic vesicles with the presynaptic membrane and, hence, neurotransmitter release and synaptic neurotransmission [1]. BoNTs are heterodimeric proteins that consist of a heavy and a light chain. The heavy chain mediates the uptake of the exotoxin into the target cells as well as the translocation of the light chain of toxin into the cell’s cytosol, where the highly specific protease activity of the small subunits cleaves and inactivates so-called SNARE (soluble N-ethylmaleimide-sensitive factor attachment protein receptor) proteins that are essential for the vesicle fusion and neurotransmitter release. There are seven or more serotypes of BoNT [2,3,4,5]. These serotypes differ in the substrate specificity of their small subunits. BoNT/A, C, and E cleave SNAP-25 on the secretory vesicle. BoNT/C, in addition, cleaves syntaxin 1 at the plasma membrane acceptor site. BoNT/B, D, F, and G cleave synaptobrevin on the vesicle [1]. With their heavy chain, the different serotypes bind to different protein receptors to enter the neurons. While BoNT/A, D, E, and F bind to synaptic vesicle protein 2 (SV2), BoNT/B, G, and DC use synaptotagmin I and II as protein receptors. In addition, all BoNT serotypes require gangliosides as co-receptors [6].

Despite their high toxicity, after local injection of small quantities, BoNTs can be used to treat a number of ailments caused by nervous hyperactivity, such as local spasms, hyperhidrosis, sialorrhea, urinary bladder dysfunction, and chronic pain [7,8,9,10]. In addition, BoNTs are widely used in aesthetic medicine [11]. Two serotypes, complexed BoNT/A and BoNT/B, are currently approved for treatment [12].

BoNTs are purified from bacterial cell culture supernatants for medical use. During the purification process, proteins that impact activity are removed [13] and variable amounts of the toxin are inactivated. To determine the activity of each single batch, activity assays are needed that faithfully monitor all steps of the toxin’s action, namely the uptake into the cell, the release of the small subunit into the cytoplasm, and the cleavage of the target protein, each of which might be impaired by partial denaturation of the BoNT protein [14].

Although a few alternative assay methods are already in place [15,16], the mouse lethality assay used to be the gold standard and is still a frequently used method to determine BoNT activity [17]. In this assay, the dose is determined at which 50% of the injected animals die of asphyxia caused by the flaccid paralysis of respiratory muscles. Apart from the ethical concerns related to this assay, it does not account for species-specific variations in the activity of different BoNT serotypes. Thus, whereas BoNT/A and BoNT/B display similar activities per mg toxin in the mouse lethality assay, about 25-fold to 100-fold higher concentrations of BoNT/B than BoNT/A are required in humans to produce similar treatment effects [18]. The reason is an amino acid sequence difference between the human and murine synaptotagmin II, the high-affinity protein receptor for BoNT/B [19,20]. An F-to-L substitution at position 54 in the human variant impairs binding. The second protein receptor, synaptotagmin I, has an approximately 10-fold lower affinity for BoNT/B in rodents [21], and appears to be less abundant on the principal target of BoNT, the motor neurons. Human synaptotagmin I binds BoNT/B with an KD > 20 µM [22]. Thus, a test system based on human cells would be more appropriate for activity determination in humans.

Currently, cell-based activity assays that determine the cleavage of the BoNT target proteins after exposure of intact cells to BoNT are the best alternatives to the mouse-lethality assay. A major disadvantage of these tests is that they determine the rate of target protein cleavage in cell lysates by ELISA tests that are based on neo-epitope-specific antibodies for the detection of the cleavage site and, hence, have an extremely narrow serotype specificity [14,16]. Recently, we devised a functional cell-based assay that determines BoNT activity by the inhibition of the stimulus-dependent release of a tagged reporter enzyme that was sorted into the neurosecretory vesicles and released together with the neurotransmitter [23,24]. Here, this test system is used to compare the activity of BoNT/A, BoNT/B, and a mutant BoNT/B (BoNT/B-MY), in which two amino acid substitutions in the toxin’s heavy-chain confer enhanced affinity to human synaptotagmin II [22].

## 2. Results

### 2.1. Inhibition of Luciferase Release by Natural BoNTs

**Figure 1:**
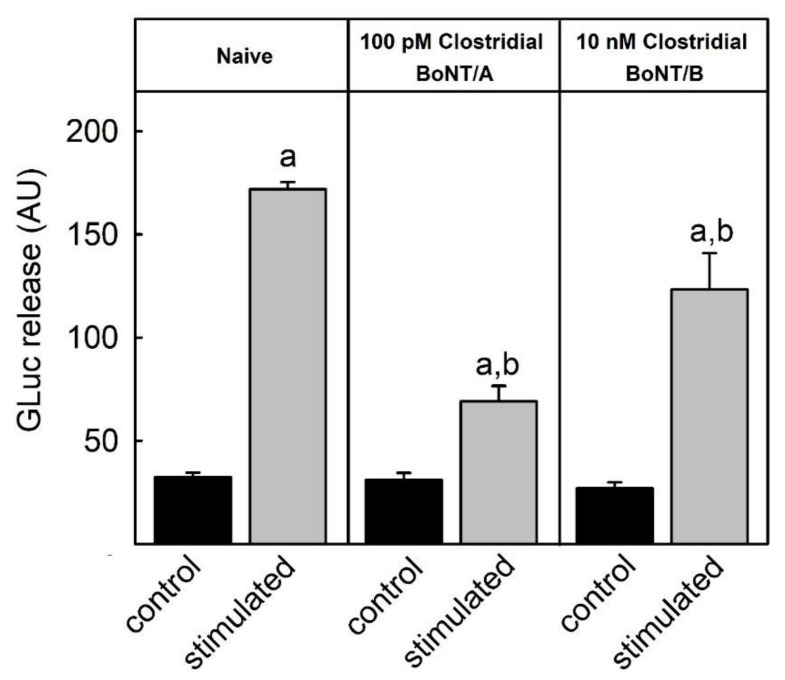
Inhibition of stimulus-dependent reporter luciferease release from SIMA-hPOMC1-26-Gluc cells by natural BoNT/A and BoNT/B. SIMA-hPOMC1-26-Gluc cells were cultured and differentiated, as detailed in the methods section. After incubation with the indicated concentrations of botulinum toxins purified from *C. botulinum* (miprolab) for 48 h, remaining toxin was removed, and cells were incubated for 3 min either in a Na+-containing control or a K+-containing depolarizing stimulation buffer. The activity of Gaussia luciferase released was determined in the cell culture supernatants. Values are means ± SEM of at least 3 independent experiments performed in triplicate. Statistics: Student’s *t*-test for unpaired samples. a: significantly different from non-stimulated control; b: significantly different from respective condition in absence of toxin; *p* < 0.05.

Previously, it was shown that BoNT/A and BoNT/C efficiently inhibited the stimulus-dependent luciferase-release from SIMA-hPOMC1-26-Gluc cells at very low picomolar concentrations while much higher nanomolar concentrations of BoNT/B were needed to obtain a partial inhibition [23]. To corroborate this finding, additional experiments with a very high concentration of BoNT/B were performed (Figure 1). Natural BoNT/A isolated from *clostridium botulinum* at a concentration of 100 pM inhibited the stimulus-dependent luciferase release by about 75%. Higher concentrations did not inhibit the release to a larger extent (not shown). At the same concentration, natural BoNT/B did not inhibit stimulus-dependent luciferase activity (not shown). By contrast, at a concentration of 10 nM, BoNT/B significantly inhibited the stimulus-dependent luciferase release by about 30% only. Higher toxin concentrations were not tested.

### 2.2. Dose Response of Recombinant Toxins

**Figure 2:**
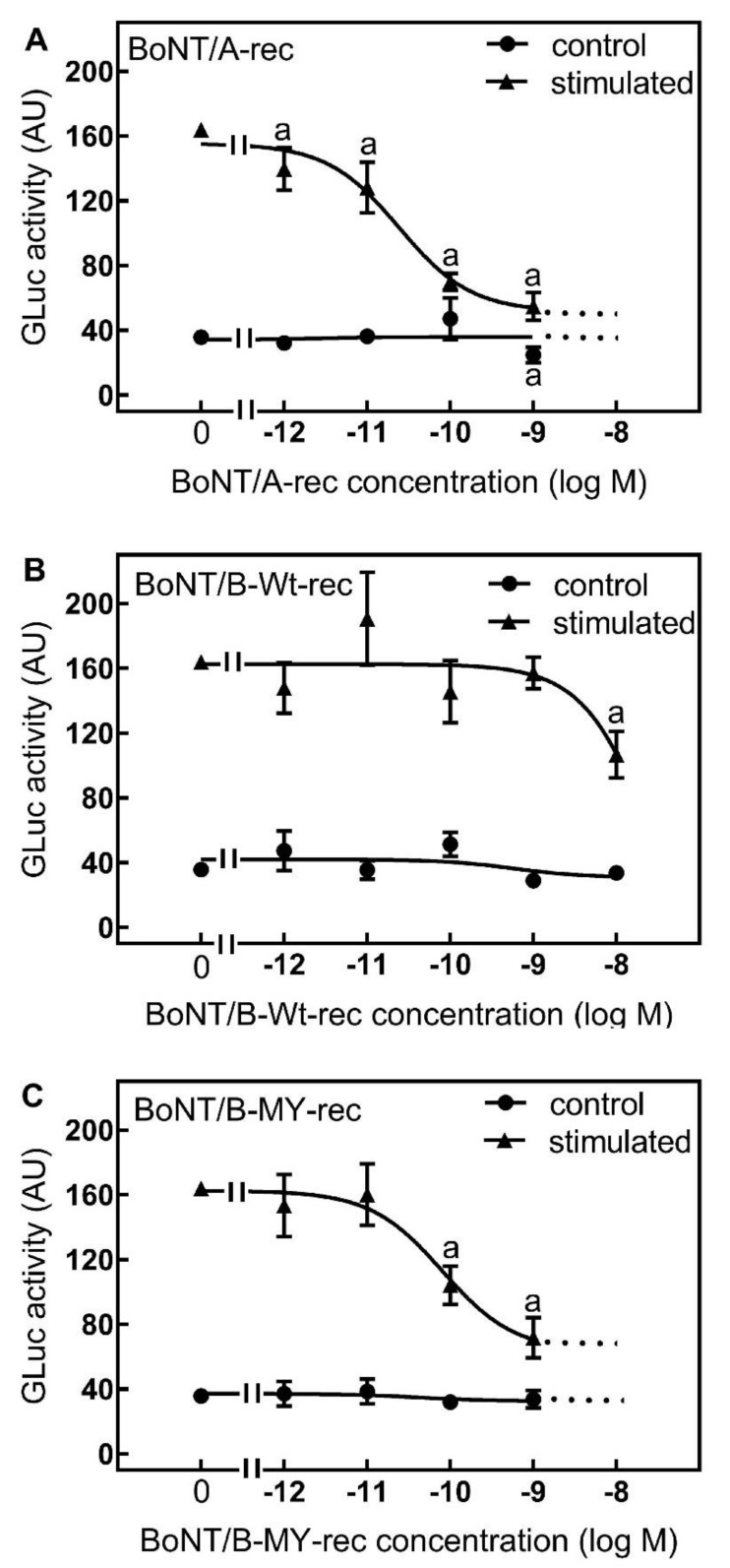
Dose-dependent inhibition of stimulus-dependent reporter luciferase release from SIMA-hPOMC1-26-Gluc cells by recombinant wild-type BoNT/A (**A**), wild-type BoNT/B (**B**), and BoNT/B-MY (**C**). SIMA-hPOMC1-26-Gluc cells were cultured, differentiated, and incubated with the indicated concentrations of recombinant botulinum toxins and stimulated as detailed in the legend to Figure 1. The release of Gaussia luciferase was determined in the cell culture supernatants. Values are means ± SEM of at least 4 independent experiments performed in triplicate per assay point. Statistics: Student’s *t*-test for unpaired samples. a: significantly different from respective condition in absence of toxin; *p* < 0.05.

Similar to BoNT/A obtained from natural sources, recombinant BoNT/A inhibited the stimulus-dependent release of Gluc from SIMA-hPOMC1-26-Gluc cells with an IC50 of about 20 pM. Already at a concentration of 1 pM, it slightly inhibited the stimulation-dependent release of the luciferase (Figure 2). By contrast, recombinant BoNT/B wild type did not inhibit the stimulus-dependent Gluc release from SIMA-hPOMC1-26-Gluc cells up to a concentration of 1 nM (Figure 2). Even at a concentration of 10 nM BoNT/B merely a moderate inhibition of the luciferase release was observed. The recombinant BoNT/B-MY has an improved affinity for human synaptotagmin II and synaptotagmin I due to a double mutation E1191M/S1199Y in the BoNT/B1 heavy chain [22]. BoNT/B-MY inhibited the stimulus-dependent release of Gluc from SIMA-hPOMC1-26-Gluc cells with a similar IC50 as recombinant BoNT/A (Figure 2).

### 2.3. Expression of Receptors

**Figure 3:**
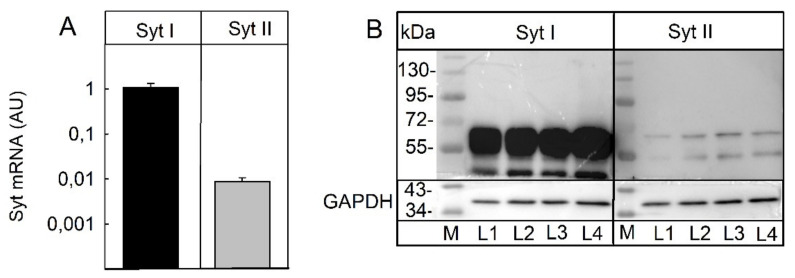
Expression of BoNT/B protein receptors on SIMA-hPOMC1-26-Gluc cells. SIMA-hPOMC1-26-Gluc cells were cultured and differentiated, as detailed in the methods section. (**A**) cDNA was reverse-transcribed from mRNA of these cells and the relative expression of synaptotagmin (Syt) I and II mRNA was determined by qPCR in comparison to GAPDH as a reference gene. (**B**) In cell lysates of these cells, Syt I and Syt II were detected with specific antibodies. The upper band at 68 kDa corresponds to the expected molecular size of both isoforms. M: marker; L: lysate.

SIMA-hPOMC1-26-Gluc cells expressed the protein receptors for BoNT/A, SV2A, and BoNT B (synaptotagmin I and II). A comparison by RT-qPCR indicated that the expression of synaptotagmin I was about 100-fold higher than that of synaptotagmin II (CT 21.3 ± 0.1 vs. 28.1 ± 0.2, mean ± SEM) (Figure 3A). To confirm RT-qPCR specificity, the product size and the cleavage of the PCR product at a product-specific restriction site checked and revealed the expected results (Supplementary Material Figure S1). Although qPCR results of different amplification protocols should not be compared directly, the results indicate a much stronger expression of synaptotagmin I than of synaptotagmin II. To confirm these results on the protein level, Western blot analysis with specific antibodies against synaptotagmin I and II was performed. The synaptotagmin I antibody recognized a very strong band at 68 kDa, a weaker band immediately below this band, a band at 48 kDa, and several fainter bands of lower apparent molecular masses (Figure 3B). The band at 68 kDa corresponds to the expected molecular mass of synaptotagmin I. The synaptotagmin II antibody recognized one band at about 68 kDa and a large number of fainter and supposedly non-specific bands. Although a direct quantitative comparison of Western blots with different antibodies is not possible, the data suggest that, in accordance with the mRNA quantification, both synaptotagmin I and II are present in SIMA cells, yet synaptotagmin I is the prevalent BoNT/B protein receptor in these cells. The abundant presence of the protein receptor for BoNT/A and SV2A in SIMA-hPOMC1-26-Gluc cells was previously confirmed [24].

### 2.4. Uptake of Toxins into Cells

**Figure 4:**
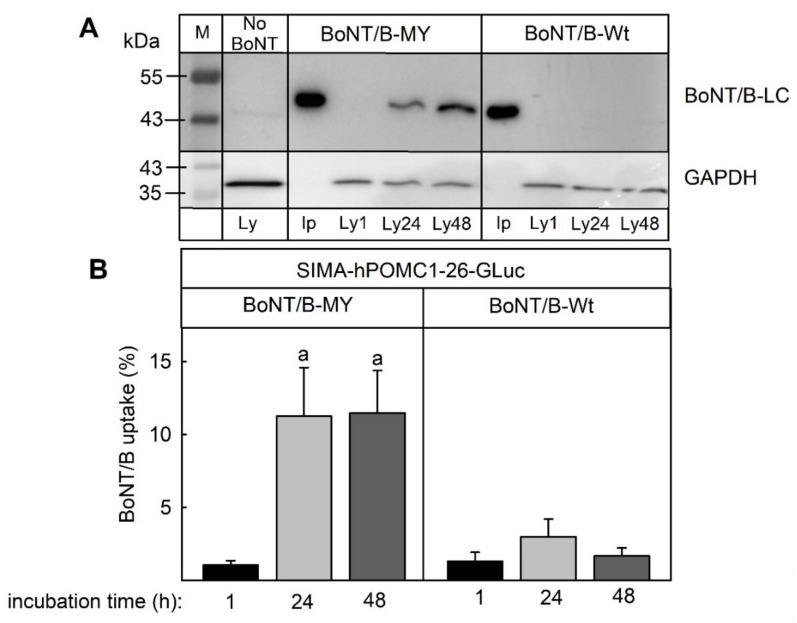
Uptake of BoNT/B-MY but not wild-type BoNT/B into SIMA-hPOMC1-26-Gluc cells. SIMA-hPOMC1-26-Gluc cells were cultured and differentiated, as detailed in the methods section and then incubated with 1 nM BoNT/B or BoNT/B-MY for the times indicated. (**A**) The concentration of the BoNT/B light chain was determined by Western blot in the medium at 0 h (input, Ip) and in cell lysates after 1, 24, and 48 h (Ly 1, Ly24, Ly48). GAPDH served as loading control. (**B**) The fraction of light chain compared to input was quantified by densitometric analysis of the western blots. Values are means ± SEM of 8 independent experiments. Statistics: Student’s *t*-test for unpaired samples. a: significantly different from 1 h incubation, *p* < 0.05.

To determine the difference in the functional interaction of wild-type and mutant BoNT/B, the cellular uptake was determined. Cells were incubated with 1 nM BoNT/B-wt or BoNT/B-MY for 1, 24, or 48 h. The concentration of the BoNT/B light chain was determined by Western blot in the input medium and cell lysates. After 1 h, neither of the toxins were detectable within the cell (Figure 4). While almost no BoNT/B-wt entered the cell after 24 or 48 h, more than 10% of the input dose of BoNT/B-MY was detected in the cell lysates, indicating that the genetically modified toxin, in contrast to the wild-type toxin, was taken up efficiently by the cells.

## 3. Discussion

The current study showed that the recently established SIMA-cell-based luciferase release assay for the determination of botulinum toxin potency can overcome the problem of species-specific variations in botulinum toxin activity: While BoNT/A inhibited the stimulation-dependent release of the luciferase from the reporter cells already at pM concentrations, BoNT/B, which is known to be much less potent in humans than in rodents [18], inhibited the stimulus-dependent reporter release only at a concentration of 10 nM (Figure 1 and Figure 2). The low activity of BoNT/B in the reporter release assay was most likely a result of the low affinity of BoNT/B for the human protein receptors for BoNT/B uptake (synaptotagmin II and synaptotagmin I). In in vitro assays with recombinant proteins, the KD for both receptors for the BoNT/B heavy chain has been reported to be above 20 µM, whereas the KD of the murine synaptotagmin II was in the range of 130 nM. Murine synaptotagmin I had a 10-fold lower affinity, but still a higher affinity than human synaptotagmin I. Consequently, within 48 h, no detectable amounts of BoNT/B were taken up by the SIMA reporter cells (Figure 4) that expressed both synaptotagmin II and, to a larger extent, synaptotagmin I (Figure 3). By contrast, a mutant BoNT/B, in which two amino acids had been replaced to increase the binding to human synaptotagmin I and II named BoNT/B-MY [22,25] inhibited the stimulus-dependent reporter release with a similar IC50 as BoNT/A (Figure 2) and was readily taken up into the SIMA reporter cells (Figure 4). Thus, in contrast to in vitro or in vivo assays that determine the inhibition of muscle contraction of wild-type mice [26] which react to BoNT/B and BoNT/B-MY with similar sensitivity, the SIMA reporter cell assay distinguished between the low potency of wild-type BoNT/B and the higher potency of BoNT/B-MY, and hence, more closely reflected the human-relevant potencies.

For the same reasons, the current assay might also be better suited than another assay proposed as a replacement method, the BINACLE assay. This assay is based on the principle that botulinum toxins are immobilized using protein receptors as a bait and, after subsequent activation of the toxin by chemical reduction, cleavage of the respective substrate peptide. Since the BINACLE assay uses murine SytII [27], it would supposedly reflect the higher affinity of the murine receptor for BoNT/B and, most likely, would not be able to detect the difference in the potencies of wild-type BoNT/B and BoNT/B-MY that is relevant for the choice of the correct dose in the treatment of human diseases.

Notably, BoNT/B can use both SytI and SytII to enter cells. SytI was the more prevalent protein receptor in SIMA-hPOMC1-26-GLuc cells. This is an important caveat, since it has been shown in mice that the main route of entry of BoNT/B into cells may differ between different tissues. Thus, the flaccid paralysis of skeletal muscle appeared to be mainly dependent on the entry of BoNT/B into motor neurons by SytII whereas the entry of the toxin into parasympathetic neurons that innervate smooth muscle cells in the urinary bladder was predominantly mediated by SytI. If in humans a similar tissue specificity for BoNT/B uptake by SytI and SytII exists, the current assay might underestimate the potency for the action in skeletal muscle [28].

The reporter cells appeared equally suited to test natural toxins, which contain additional proteins that might affect uptake, and recombinant toxins, which are devoid of the accessory proteins. This is in accordance with results from previous studies that showed that the test system detected non-purified BoNT/A with similar sensitivity, as toxins that were highly purified for therapeutic use [23,24]. Nevertheless, the comparatively low sensitivity of this system might be a limitation to the broad applicability of this assay. Thus, assuming that one mouse lethality unit corresponds roughly to a 1 pM toxin solution, the mouse lethality assay is about 10-fold more sensitive than the current SIMA reporter cell-based assay. In addition, it was shown that the sensitivity of human motor neurons derived from induced pluripotent stem cells have an approximately 200-fold higher sensitivity for the BoNT/A-induced SNAP25 cleavage than SIMA cells [29]. Similarly, a very recently established test system that determines the botulinum toxin activity in motor neurons differentiated from induced pluripotent stem cells that express a split luciferase substrate for BoNT/A was shown to detect BoNT/A with an approximately 10-fold higher sensitivity than the current assay ([30] published as pre-print). A drawback of this latter assay that detected the cleavage of the substrate proteins by immunological techniques in comparison to the current assay is the limited versatility, because a specific cell line or specific (neoepitope-specific) antibodies are needed for each serotype, whereas the current assay determines a functional endpoint that is common to all serotypes. Thus, besides BoNT/A and genetically modified BoNT/B, it also detected BoNT/C with similar sensitivity [23].

## 4. Conclusions

In conclusion, the SIMA-hPOMC1-26-GLuc-cell-based assay may represent a versatile tool to determine the activity of natural and genetically modified BoNT serotypes. Furthermore, the increased activity of the genetically modified BoNT/B-MY variant was due to an increased uptake by SIMA-hPOMC1-26-GLuc cells.

## 5. Materials and Methods

### 5.1. Materials

All chemicals were purchased from commercial sources indicated throughout the text. Oligonucleotides were custom-synthesized by Eurofins Operon (Ebersberg, Germany) or Biolegio (Nijmegen, The Netherlands). RPMI 16040 medium, heat-inactivated fetal calf serum (FCS), stable L-alanyl-L-glutamine, nonessential amino acids, penicillin, and streptomycin were from PAN-Biotech, Aidenbach, Germany. B27 and N2 supplement were purchased from Life Technologies, Darmstadt, Germany.

### 5.2. Cell Culture

The generation of the stably transfected neuroblastoma cell line SIMA-hPOMC1-26 GLuc has been described previously [24]. Non-transfected SIMA cells were originally from DSMZ, (Braunschweig, Germany). Cells were cultured in RPMI 16040 medium, supplemented with 10% (*v*/*v*) heat-inactivated fetal calf serum (FCS), 2 mM stable L-alanyl-L-glutamine, and penicillin (100 U/mL)/streptomycin (100 µg/mL) as antibiotics.

### 5.3. Luciferase Release from BoNT/X-Treated Cells

For release experiments, SIMA-hPOMC1-26-GLuc cells were differentiated in poly-l-lysine-coated 96-well plates (5 × 104 cells/well) with differentiation medium (RPMI supplemented with 1 × B27 supplement, 1 × N2 supplement, 2 mM L-alanyl-L-glutamine, 1 mM non-essential amino acids, 10 mM 4-(2-hydroxyethyl)-1-piperazineethanesulfonic acid (HEPES), and penicillin (100 U/mL)/streptomycin (100 µg/mL) for 48 h. Cells were then incubated with different concentrations of BoNT/A or BoNT/B (Miprolab, Göttingen, Germany) or recombinant BoNT/A, BoNT/B-wt, or BoNT/B-MY mutant (Ipsen, Abingdon, UK) in a differentiation medium for 48 h. Subsequently, cells were pre-incubated with 100 µL of fresh medium for 10 min at 37 °C. The medium was aspirated, and GLuc release was stimulated with 100 µL/well control (20 mM Hepes pH 7.4, 136 mM NaCl, 4.7 mM KCl, 1.25 mM CaCl_2_ and 1.25 mM MgSO_4_) or depolarization buffer (20 mM Hepes pH 7.4, 40.7 mM NaCl, 100 mM KCl, 1.25 mM CaCl_2_ and 1.25 mM MgSO_4_) for 3 min at 37 °C. The supernatant was transferred into reaction vials and centrifuged at 100× *g* for 3 min to remove detached cells. To determine GLuc activity, 20 µL of the supernatant was mixed with 100 µL of luciferase substrate solution, and the luminescence was measured using Fluostar Optima (BMG Labtech, Ortenberg, Germany). GLuc release was normalized to GLuc activity in the remaining lysed cells, and the mean of GLuc activity in untreated control and stimulated cells was set to 100% (AU).

### 5.4. BoNT/B Uptake Assay

For BoNT/B uptake experiments, SIMA-hPOMC1-26-GLuc cells were differentiated for 48 h, as described in the release experiment section. Subsequently, cells were incubated in triplicate with 1 nM BoNT/B-wt or BoNT/B-MY in 100 µL of differentiation medium/well for 1 h, 24 h, or 48 h at 37 °C. Cells were washed three times with differentiation medium and lysed in 50 µL/well Lämmli sample buffer (80 mM Tris/HCl pH 6.8, 2% (*w*/*v*) SDS, 5% (*w*/*v*) glycerol, 0,025% *w*/*v* bromphenol blue, and 5% (*v*/*v* 2-mercatoethanol). Lysates from triplicate wells were pooled and incubated at 95 °C for 10 min. Insoluble material was removed by centrifugation (10,000× *g*, 15 min, 4 °C). Proteins from lysates or BoNT/B-wt/my input were resolved by SDS-PAGE and transferred to a polyvinylidene difluoride (PVDF) membrane. Membranes were blocked in 5% non-fat dry milk in 20 mM Tris, 136 mM NaCl, and 0,1% (*v*/*v*) TWEEN 20 (Polyoxyethylenesorbitan monolaurate, TBS/Tween) for 1 h at room temperature. They were then incubated with an anti-BoNT/B light chain antibody (Life technologies, Darmstadt, Germany, PA-5-47737, 1:1000) in TBS/Tween containing 5% bovine serum albumin overnight at 4 °C and a horseradish peroxidase-conjugated anti-sheep antibody for 2 h at room temperature. Visualization of immune complexes was performed using chemiluminescence reagent Clarity Western ECL (Bio-Rad laboratories, Feldkirchen, Germany).

### 5.5. Syt Western Blot

SIMA-hPOMC1-26-GLuc cells were lysed in Lämmli sample buffer (80 mM Tris/HCl pH 6.8, 2% (*w*/*v*) SDS, 5% (*w*/*v*) glycerol, 0.025% *w*/*v* bromphenol blue, and 5% (*v*/*v* 2-mercatoethanol) homogenized by sonication. Insoluble material was removed by centrifugation (10,000× *g*, 15 min, 4 °C). Western blot was performed as described in the BoNT/B uptake assay, using anti-Syt antibodies (Syt I: Proteintech, St-Leon-Rot, Germany, 14511-1 and SytI/II: Santa Cruz Biotechnology, Heidelberg, Germany, sc-393392) and a horseradish peroxidase-conjugated anti-rabbit antibody.

### 5.6. Real-Time RT-PCR

The total RNA from differentiated SIMA-hPOMC1-26-GLuc cells was isolated using the peqGold Total RNA Kit (Peqlab, Darmstadt, Germany). Then, 1–2 µg of the total RNA was reverse-transcribed into cDNA using an oligo dT as a primer and an M-MuLV Reverse Transcriptase (Thermo Scientific, Germany). Hot-start real-time PCR for the quantification of each transcript was carried out using 2 × Maxima SybrGreen qPCR mix (Life technologies), 0.25 µM of each primer, and 2.5 µL–5 µL of cDNA, which was diluted 1:10. PCR was performed with an initial enzyme activation step at 95 °C for 10 min, followed by 42 cycles of denaturation at 95 °C for 30 sec, annealing at 57 °C for 30 sec, and an extension at 72 °C for 1 min in a real-time DNA thermal cycler (CFX96™, 10 µL reaction volume, Bio-Rad). The oligonucleotides used are listed in Table 1. The expression levels of SytI/SytII were calculated relative to GAPDH as a reference gene. Because of the low SytII mRNA expression level, Syt II qPCR was performed with three different primer combinations.

## 6. Patents

No patents result from this study.

## Figures and Tables

**Table 1:** Oligonucleotide primers used for real-time qPCR.

Gene	Forward	Reverse
GAPDH	5′-TGATGACATCAAGAAGGTGG	5′-TTACTCCTTGGAGGCCATGT
SytI	5′-ACCATTGAGGAAGAGGCAGC	5′-TTACTCCTTGGAGGCCATGT
SytII-1	5′-CATTGGACCCGTGGACAACT	5′-AGAACGCCCACAGTAAGCTG
SytII-2	5′-CATTGGACCCGTGGACAACT	5′-GGCCATCACCAGAGTTTTGC
SytII-3	5′-TCAGCTTACTGTGGGCGTTC	5′-GGCCATCACCAGAGTTTTG

Accession numbers for the genes were: GAPDH (AB062273), SytI (NM_005639.2), and SytII (NM_177402.4).

## Data Availability

Original data and Excel files are available on request.

## Supplementary Materials

The following are available online at https://www.mdpi.com/article/10.3390/toxins14010065/s1, Figure S1: Confirmation of RT-Syt I/II qPCR specificity.
