# Supplementary material for: Discrimination of the Activity of Low-Affinity Wild-Type and High-Affinity Mutant Recombinant BoNT/B by a SIMA Cell-Based Reporter Release Assay
Source: Toxins (Basel). 2022 Jan 17;14(1):65. doi: 10.3390/toxins14010065 (PMC8780439; doi:10.3390/toxins14010065)
Supplement: Supplementary file 1 [file toxins-14-00065-s001.zip › toxins-1470624-supplementary.pdf]

toxins

Supplementary Materials: Discrimination of the Activity of Low-Affinity Wild-Type and High-Affinity Mutant Recombinant BoNT/B by a SIMA Cell-Based Reporter Release Assay

Frank Neuschäfer-Rube, Andrea Pathe-Neuschäfer-Rube and Gerhard P. Püschel

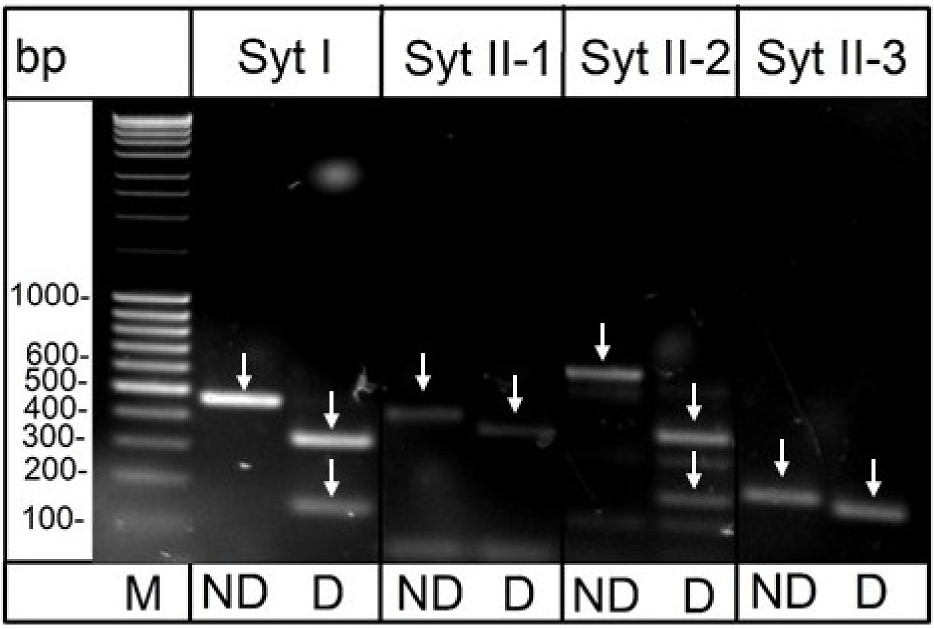

Figure S1. Confirmation of RT-Syt I/II qPCR specificity. PCR products amplified by Syt I/II RT-qPCRs were digested with restriction enzymes at internal recognition sites. Non digested (ND) and digested (D) PCR products were separated by agarose electrophoresis and stained with ethidiumbromide. The size of DNA bands was calculated relative to the MassRuler DNA Ladder (M). Expected size of the DNA bands (marked by arrows) were: SytI : ND, 459 bp, D (Eco0109I): 322 bp+137 bp; SytII-PCR1: ND, 414 bp, D (PstI) 363 bp + 51 bp (not visible), SytII-PCR2: ND, 607 bp, D (PstI) 363 bp + 190 bp + 54 bp (not visible), SytII-PCR3: ND, 213 bp, D (PstI) 190 bp + 24 bp (not visible).
